# Highfield imaging of the subgenual anterior cingulate cortex in uni- and bipolar depression

**DOI:** 10.3389/fpsyt.2024.1462919

**Published:** 2024-10-11

**Authors:** Frederik Buchholz, Martin Meffert, Pierre-Louis Bazin, Robert Trampel, Robert Turner, Peter Schönknecht

**Affiliations:** ^1^ Department of Psychiatry and Psychotherapy, University Hospital Leipzig, Leipzig, Germany; ^2^ Full Brain Picture Analytics, Leiden, Netherlands; ^3^ Department of Neurophysics, Max Planck Institute for Human Cognitive and Brain Sciences, Leipzig, Germany; ^4^ Out-patient Department for Sexual-therapeutic Prevention and Forensic Psychiatry, University Hospital Leipzig, Leipzig, Germany; ^5^ Department of Psychiatry, Psychotherapy and Psychosomatic, Saxon State Hospital Altscherbitz, Leipzig, Germany

**Keywords:** major depressive disorder, bipolar disorder, gray matter volume (GMV), subgenual ACC, psychopathology

## Abstract

**Background:**

The subgenual Anterior Cingulate Cortex (sgACC), as a part of the Anterior Cingulate Cortex and the limbic system plays a crucial role in mood regulation. Previous structural and functional brain imaging studies of the sgACC have revealed alterations of Gray Matter (GM) volumes and Blood Oxygenation Level Dependent signals (BOLD) in patients with Major Depressive Disorder (MDD) and Bipolar Disorder (BD), suggesting potential biomarker traits for affective disorders.

**Method:**

In this study we investigated the gray matter volume of the sgACC in 3 different patient groups: 40 MDD patients, of which 20 were medicated (MDDm) and 20 were unmedicated (MDDu), and 21 medicated BD patients, and compared them with 23 healthy volunteers. We examined GM volume alteration using high-resolution 7T Magnetic Resonance Imaging (MRI) which produced quantitative maps of the spin-lattice relaxation time (T1). T1 maps provide high contrast between gray and white matter, and at 7 Tesla voxels with submillimeter resolution can be acquired in a reasonable scan time. We developed a semi-automatic segmentation protocol based on refined landmarks derived from previous volumetric studies using quantitative T1 maps as raw input data for automatic tissue segmentation of GM, WM and CSF (cerebrospinal fluid) tissue. The sgACC ROI was then superimposed on these tissue probability maps and traced manually by two independent raters (F.B., M.M.) following our semi-automatic segmentation protocol. Interrater reliability was calculated on a subset of 10 brain scans for each hemisphere, showing an Intra-Class Correlation coefficient (ICC) r = 0.96 for left sgACC and r = 0.84 for right sgACC respectively. In summary, we have developed a reproducible and reliable semi-automatic segmentation protocol to measure gray matter volume in the sgACC. Based on previous findings from meta-analyses on morphometric studies of the sgACC, we hypothesized that patients with MDD would have lower gray matter sgACC volumes compared to healthy subjects.

**Results:**

Post-hoc analysis revealed smaller subgenual volumes for the left hemisphere in both the medicated (MDDm) and non-medicated (MDDu) group versus healthy controls (p = .001, p = .008) respectively. For the right hemisphere, the (MDDu) and BD group exhibited significantly lower subgenual volumes than healthy controls (p < .001, p = .004) respectively.

**Conclusion:**

To our knowledge, this is the first morphometric MRI study using T1 maps obtained in high-resolution 7 Tesla MRI to compare MDD and BD patients with healthy controls.

## 1 Introduction

The human subgenual Anterior Cingulate Cortex (sgACC), a part of the rostral anterior cingulate cortex (ACC) located just below the genu of the corpus callosum (CC), is considered to be a gateway for fronto-limbic networks mainly involved in affective disorders, both major depressive disorder (MDD) and bipolar disorder (BD). The ACC can be divided into different subregions based on functional and histological differences, including receptor distribution (1–3). (4) found reduced numbers and densities of glia in MDD and BD in the subgenual part of Brodmann area 24 (BA24sg), but no change in neuronal numbers or neuronal density in post-mortem analysis. Similar results for MDD could be shown by (5), who found evidence for glia alterations and a small decrease in neuronal size while (6) reported reduction in neuronal soma size and increased neuronal density in the ACC. (7, 8) reported predominantly neuronal density reductions in BD patients. Meta-analyses conducted on structural magnetic resonance imaging (MRI) studies with region-of-interest (ROI) approach revealed reduced ACC volumes in both right and left ACC in patients with MDD compared to healthy controls (9, 10) or as reported by (11), mainly in antidepressant-free patients compared to healthy controls. Further evidence for reduction of gray matter in sgACC area by means of using Voxel-based Morphometry (VBM) analyses were shown by (12, 13). Attempts to define the functional connectivity of the sgACC using tractography analysis in Diffusion Tensor Imaging (DTI) have suggested that the sgACC is highly connected to the amygdala, hypothalamus, nucleus accumbens and orbitofrontal cortex (14), (15). DTI studies on BD patients revealed an increased connectivity between left sgACC and left amygdalohippocampal complex (16), while (17) observed a cluster of decreased fractional anisotropy in right sgACC in BD patients. (18) showed in medication-naive patients with recurrent MDD a significant decrease in 5-HT1B receptor binding potential in the subgenual ACC (17% between-group difference), a serotonin receptor subtype with a potential role in the development of depressive Symptoms. The ACC and in particular the sgACC show alterations in functional studies in affective disorders. (19) found decreased blood flow and glucose metabolism in the sgACC as well as reduction in the gray matter in the left sgACC. Reduced blood flow in the sgACC was also shown in Positron Emission Tomography (PET) by (20), while functional MRI (fMRI) studies have reported hyperactivity in rostral ACC (21, 22) In addition, baseline hyperactivation in the sgACC was found to be predictive of treatment resistance and poor treatment outcomes (23). (24) observed elevated connectivity between the sgACC and the insula and also between sgACC and the amygdala in drug-naïve MDD patients. Increased connectivity between sgACC and dorsolateral frontal cortex (DLPFC), which correlates positively with the severity of depressive symptoms, was observed by (25) and the sgACC has been since an indirect target for repetitive Transcranial Magnetic Stimulation (rTMS) protocols in depression (26), albeit shown to account only for a modest treatment outcome in its current state (27). Studies in Electroconvulsive Therapy (ECT), one of the most effective treatments for depression, particularly in treatment resistant depression, also found higher BOLD signal fluctuations (fALFF) in the sgACC area at baseline in depressed patients, which decreased over the course of ECT. In addition, the authors note that connectivity of sgACC with the hippocampus and other cortical areas was significantly reduced, suggesting that the antidepressant effect of ECT may be mediated by the downregulation of sgACC activity and connectivity (28). Finally, in clinical treatment the posteriorly adjacent subcallosal ACC is already an important site for deep brain stimulation (DBS) in the treatment of refractory depression (29, 30).

The aim of this study is to investigate gray matter volume among patients with major depressive disorder (MDD) and bipolar disorder (BD) (19), while benefiting from the higher resolution that 7T MRI provides over 1.5T or 3T MRI scanning.

We kept in line with previous clinical segmentation protocols using a region-of-interest (ROI) approach to measurement of the sgACC (19, 31). We hypothesized that subjects with MDD would have lower mean volumes in the subgenual ACC region compared to healthy controls. In addition, we applied the segmentation protocol to quantitative T1 maps obtained with the MP2RAGE sequence (32), a sequence in which contrast is obtained with two adiabatic inversion pulses and a low flip-angle gradient echo signal acquisition. In brain applications, T1 mapping sequences may have an advantage over other MRI sequences as they separate quantitative T1 from other MR parameters to characterize tissue properties such as myelin, iron, and water content (33). We applied our segmentation protocol to a sample of 40 MDD patients, of whom 20 were medicated (MDDm), 20 were unmedicated (MDDu), together with 21 medicated BD patients, and compared them with 23 healthy volunteers to examine for GM (gray matter) volume alteration. We hypothesized that subjects with MDD would have lower mean volumes in the subgenual ACC region compared to healthy controls.

## 2 Materials and methods

### 2.1 Study design and participants

The study design comprised four study groups with at least 20 subjects divided by diagnosis, ensuring sufficient test power (1-b = 0.80) for large-sized effects with an alpha error rate of 5% in a one-way ANOVA with fixed effects and four groups (34). Psychiatric inpatients and outpatients in a depressed mood state, aged 18 to 65 years (mean age 38 ± 12 years), Caucasian, were recruited through the University Hospital Leipzig. Two patient groups were medicated (MDDm and BD) while one MDD group (MDDu) received no psychopharmacological medication for at least three months prior to MRI. The study was approved by the local ethics committee of the university Leipzig, and written informed consent was obtained from all participants prior to the investigation, according to the declaration of Helsinki. From 107 subjects initially included in the study, 91 completed the Structured Clinical Interview for DSM-IV (SCID) (50) to establish axis I lifetime diagnoses, and 87 received a complete 7T MRI. Diagnoses were validated after 2–4 years, based on long-term clinical records, which required a switch of diagnosis for one patient (female, 20 years) from recurrent MDD to bipolar I disorder. This resulted in 84 subjects (20 MDDu, 20 MDDm, 21 BD, 23 healthy controls) being entered for volumetric analysis. Diagnosis of MDD according to the criteria of DSM-IV was assessed for each patient by clinical evaluation of a senior physician; symptom severity was measured using the structured interview SIGH-D17/IDS-C30 for Hamilton Rating Scale for Depression (HRSD), Inventory of Depressive Symptomatology (IDS), Beck Depression Inventory II (BDI-II) (35) and Bech Rafaelsen Melancholia Scale (BRMS) (36). In addition, the duration of illness, the duration of the current depressive episode and the number of episodes were recorded (see **Table 1** below for a summary). Further information regarding demographics of the study groups and the clinical course of the patients can be found in (37). Exclusion criteria for patients were (1) a clinically significant neurological or other medical disorder (e.g. dementia or organic brain disorder, including history of traumatic brain injury), (2) current or previous addiction or substance abuse, (3) suicidality and (4) general MR imaging exclusion criteria.

**Table 1:** Comparison of the study groups regarding demographic and clinical characteristics.

	MDDm(n=20)	MDDu (n=20)	BD (n=21)	HC (n=23)	Group comparison (n=84) Test statistic	Correction for ICV (n=84) P-value
Sex (male/female)	8/12	7/13	9/12	9/14	v23, N=84 = 0.27	0.97
Age	36.2 ± 12.8	42.9 ± 10.8	39.3 ± 12.0	36.0 ± 12.8	F_3, 80_ = 1.47	0.23
Intracranial volume (liter)	1.54 ± 0.16	1.52 ± 0.15	1.53 ± 0.12	1.55 ± 0.12	F_3, 80_ = 0.34	0.80
Beck Depression Inventory II	26.4 ± 10.2	21.6 ± 10.6	22.5 ± 13.4	n.a.	F_2, 58_ = 0.97	0.38
Bech–Rafaelsen Melancholia Scale	16.3 ± 4.9	14.8 ± 7.9	18.6 ± 9.0	n.a.	F_2, 36_ = 1.03^w^	0.37
Hamilton Rating Scale for Depression	17.5 ± 6.9	16.2 ± 9.7	19.5 ± 8.9	n.a.	F_2, 58_ = 0.78	0.46
Inventory of Depressive Symptomology	31.3 ± 12.4	30.9 ± 13.9	33.0 ± 15.9	n.a.	F_2, 58_ = 0.13	0.88
Years since 1^st^ episode	7.6 ± 10.4	13.3 ± 10.5	15.9 ± 10.6	n.a.	F_2, 57_ = 3.20	0.048*

*p ≤.05, ^A^AN(C)OVA statistics, MDDm, Major Depressive disorder (medicated); MDDu, Major Depressive disorder (unmedicated); BD, Bipolar disorder; HC, healthy controls; ICV, Intracranial volume. The p values listed in the tables are not corrected for multiple testing using Bonferroni correction, n.a. not available, ^w^Welch Statistic.

### 2.2 Image acquisition and preprocessing

All Magnetic Resonance (MR) images were acquired using a 7T whole-body scanner (MAGNETOM 7T, Siemens Healthineers, Erlangen, Germany) as well as a 24 channel-NOVA coil (Nova Medical, Inc., Wilmington, MA, USA). A 3D Magnetization-Prepared 2 Rapid Acquisition Gradient Echoes sequence (MP2RAGE) (32) and a TR-FOCI pulse for inversion were applied (38). T1 maps were obtained using the following parameters: repetition time TR=8,25 s, inversion times Tl1/Tl2 = 1 s/3,3 s, flip angles (a1/a2) = 7°/5°, echo time (TE) = 2.51 ms, receiver bandwidth (BW) = 240 Hz/Px, 1 average. A field of view (FOV) of 224 mm x 224 mm x 168 mm and an imaging matrix of 320 x 320 x 240 resulted in a nominal acquisition voxel size of 0.7 mm isotropic. By accelerating the acquisition using parallel imaging (39) with an acceleration factor of two, a scan time of 18:02 minutes was achieved.

Nighres (40) (Version 1.4.0), a Python-based toolbox, was used for preprocessing of the uniform MP2RAGE images. The quantitative maps of the spin-lattice relaxation time (T1) were skullstripped for extracerebral tissue. Intracranial volume (ICV) was determined as the number of non-zero voxels after skull-stripping. T1 maps were then used as raw input data for automatic tissue segmentation of Tissue Probability Maps (TPM) with three tissue compartments of GM, WM and CSF for both cortical hemispheres separately, following the MGDM and CRUISE cortical segmentation pipeline in Nighres (41). A simple ridge structure filter was used to enhance sulcal CSF contrast. All images and their respective TPM were then aligned to the standard orientation of Mai et al. (42) using LIPSIA software (43) with shifted linear interpolation (44).

### 2.3 Defining anatomical region of the sgACC ROI

**Figure 1:**
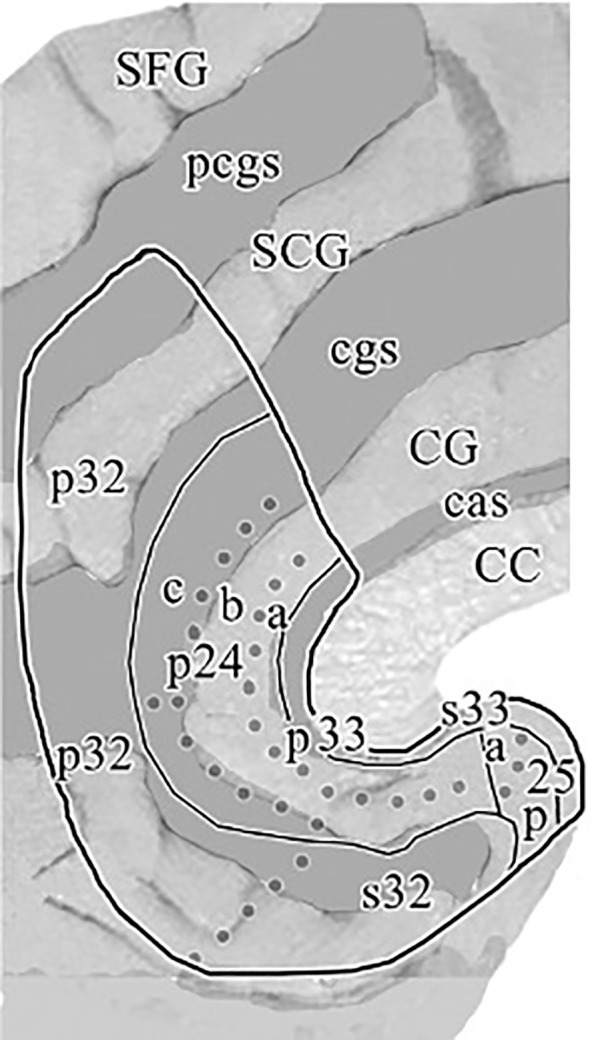
“Flat map” of the medial surface of the human ACC showing the distribution of subregions and areas from a histological assessment based on a flattening procedure to allow a more accurate visualization of the examined cingulate areas as described in the text. The sulcal depths are noted with a homogeneous gray color. CC, corpus callosum; CG, cingulate gyrus; SCG, superior cingulate gyrus; SFG, superior frontal gyrus; cas, callosal sulcus; cgs, cingulate sulcus; pcgs, paracingulate sulcus. Reprinted with permission from 'Cytology and Receptor Architecture of Human Anterior Cingulate Cortex' by Palomero-Gallagher et al., doi 10.1002/cne.21684, Copyright © 2008 Wiley-Liss, Inc.

In previous clinical applications, (19) defined the subgenual part of Brodmann’s area 24 (sg24) as the first full gyrus inferior to the Corpus Callosum (CC), that is bound anteriorly by the vertical plane through the rostral tip of the genu corporis callosi. Posteriorly it is bound by the first coronal slice, in which the Capsula Interna (CI) separating the caudate from the putamen can be visualized. Furthermore, we followed the classification scheme implied in (3, 45) and defined the region of subgenual ACC primarily with reference to the boundaries depicted in the cytoarchitectonic maps for the subgenual region of Brodmann area 24a/b. As can be seen on the flat map in **Figure 1** (1), there is a further subdivision within BA24 into three subregions 24a, 24b and 24c. While 24c is found in the perigenual area, our segmentation method intended to delineate subgenual regions s24a and s24b. In accordance with previous clinical applications (19), the anterior boundary of the subgenual ACC was adopted as the first slice of cortex beneath the rostral tip of the genu corporis callosi (CC). The superior boundary of the ROI was represented by callosal sulcus (CaS) delineating it from the adjacent BA 33, which can be found as a narrow strip of cortex along the inner bank of the callosal sulcus. BA 33 was excluded from the ROI as it is merely described to be a hippocampal embryological remnant mainly assigned to interoception and pain perception domains (46). The inferior boundary of the ROI was represented by the Cingulate Sulcus (CiS), and if the CiS did not extend caudally beyond the full subgenual area, a line of its expected course was extended to ensure a reliable boundary to adjacent region BA 32. The posterior boundary as described in (19) as the last slice of cortex before the Capsula Interna (CI) becomes visible and separates the caudate from the putamen could not be reliably implemented into our segmentation protocol, as the transition of the CI becoming clearly visible often occurred throughout multiple slices. Instead, we used the vertical extension of the Anterior Parolfactory Sulcus (APS) as a posterior boundary, which was previously described by (Ono et al. (47) and reaffirmed in post-mortem analysis (48) as a practical boundary to delineate subgenual cingulate (BA24) from the posterior subcallosal area (BA25), as it was also intended in (19) method. Although the APS is estimated to be missing in about 13-20% of cases (46, 48), we found that most of the times at least part of its vertical branch could be traced and analogous to (48) method we defined the posterior boundary of our ROI as the extension between the upper end of APS and its linear extension to the Rostrum. If, in some cases, the APS was not visible at all, a vertical extension from the Rostrum was utilized as maximum posterior limit.

### 2.4 sgACC segmentation procedure

**Figure 2:**
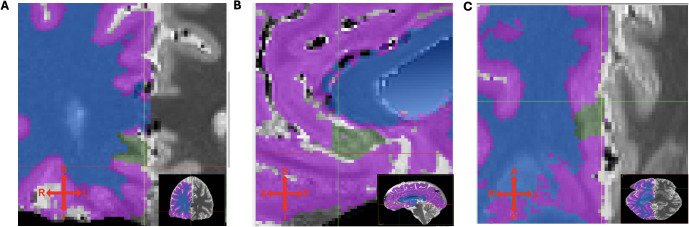
**(A)** coronal, **(B)** sagittal and **(C)** transversal view of T1 maps with segmented (green) sgACC ROI. Tissue probability maps (TPM) indicating different tissues types (for right hemisphere only): purple - GM, blue - WM, transparent - cerebrospinal fluid. Crosshair indicates superior, inferior, left and right direction.

Left and right sgACC volumes were measured according to our segmentation protocol (Document S1) using the publicly available Medical Image Processing and Visualization software (49) (MIPAV, Version 11.0.8) for interactive masking in triplanar view. Tissue probability maps (TPM) with a fixed intensity range of [0:4095] were superimposed on the native grayscale T1 images to allow clear visualization of distinct tissue compartments labeled (1) – GM, (2) - WM and transparent voxels corresponding to CSF, which allowed WM-defined tissue to be masked out before starting the segmentation procedure to prevent inadvertent segmentation of non-GM voxels. After initial anteroposterior compartmentalization of the sgACC-ROI, a rater’s task was then to mark out the sgACC GM tissue using the anatomical landmarks and automatic segmentation boundaries defined in our segmentation protocol. Segmentation was performed in coronal view, slice by slice, in anterior-posterior direction. Subsequently, remaining GM voxels within the ROI were filled out in sagittal and axial planes. Finally, all masked voxels were reviewed in all three planes by fading in and out and compared to the underlying anatomy. To assess the reliability of the semi-automatic segmentation method for sgACC gray matter tissue on high-resolution 7T MRIs, a subsample of 10 brain scans with respect to even representation of age, sex and diagnostic group were preselected and segmented by two trained independent raters (F.B., M.M.), who were blinded to subject diagnosis. Subjects’ datasets were randomly displayed either left-right mirrored or not mirrored ensuring that the rater worked on the left side of the screen during the entire segmentation procedure. There were constant working conditions regarding light, display settings, and zoom. An example of a segmented sgACC ROI in triplanar view can be seen on **Figure 2**. The intraclass correlation coefficient (ICC), calculated from 10 subjects (10 left and 10 right sgACC) for the manual segmentation of sgACC volumes resulted in a value of r = 0.96 on the left hemisphere and, r = 0.84 for right sgACC.

### 2.5 Data characteristics and statistical analysis

Statistical analyses were carried out using SPSS Statistics 29 (SPSS Inc. Released 2009. PASW Statistics for Windows, Chicago, USA). All tests were two-tailed and P-values below.05 were considered significant. Normality was tested using the Shapiro-Wilk test and was confirmed (p >.05) for all variables except for age, BRMS, age of onset, number of depressive episodes, duration of illness, and duration of the current episode. We detected two unusually large sgACC volumes, one in the left UPU group (595mm^3^) and one in the right BD group (496mm^3^). For comparison of subgroup volumes, we performed one-way analysis of covariance (ANCOVA) with sgACC volumes as the dependent variable and diagnosis (20 MDDu, 20 MDDm, 21 BD, and 23 C) as the grouping variable covaried for intracranial volume (ICV). Given high intercorrelations between the left and the right sgACC volumes (r = .35, P = .001) and in order to analyse sgACC volumes for both hemispheres separately, a univariate approach was chosen. ANCOVA results obtained under inhomogeneity of variance (Levene Statistic, P <.05), were validated by partialling out the covariates and calculating a robust Welch Statistic for the residuals. Correlations with confounding variables related to sgACC volume are presented in **Table 2**. When there was a significant main effect of diagnosis, *Post hoc* group comparisons were evaluated against a Bonferroni-adjusted α level of significance set at p <.05 to correct for multiple comparisons. Finally, we explored relationships between our volumes and clinical characteristics. Correlations with depressive, non-depressive or overall episode counts were calculated using Pearson’s r or Spearman’s q for non-normally distributed data respectively. For correlations with clinical ratings, Kendall’s τ was used to account for non-equidistant ordinal data. Homogeneity of (co)-variance was tested for all group comparisons using Brown-Forsythe test.

**Table 2:** Impact of candidate confounding variables on the sgACC volume.

	left		right	
	Test statistic	p-value	Test statistic	p-value
ICV	r = .12	0.29	r = .26	.016*
Gender	t = 1.96	0.054	t = 1.48	0.143
Age	*ρ* = -.05	0.645	*ρ* = .015	0.89

*p ≤.05, ICV, Intracranial volume, Pearson’s correlation coefficient r, Student’s t for independent t-tests, Spearman’s correlation coefficient ρ.

### 2.6 Classification-related issues

Macroscopic inter-individual differences in brain morphology, e.g. sulcal and gyral folding variability must be carefully considered to correctly assign the designated ROI to a corresponding functional Brodmann area. Particularly in the subgenual region, the cingulate sulcus (CiS) did not always extend sufficiently into the ventro-caudal direction of the ROI to serve as a reliable inferior boundary between s24 and s32. When this was the case, a line had to be traced as an approximation of its expected course to delineate s32 from our target region s24. In about 30-60% of cases, the CiS shows a double parallel distribution forming two cingulate gyri (50, 51). The inner one is defined as the main ACC, corresponding to BA 24, and the one located between the CiS and the outer pcgs (paracingulate sulcus) is considered to be the SCiG (superior cingulate gyrus) or paracingulate gyrus, corresponding to BA 32 (3, 52). Interpretation of sulcal structures became further complicated when in some cases the appearance of the intralimbic sulcus (ILS) superior and parallel to the CiS also gave the impression of a double-folded pattern. It was previously described by Vogt et al. (3) as a secondary cingulate sulcus (though not named) or by Paus et al. (51) as a shallow sulcus between the cingulate and callosal sulci. In their post-mortem analysis on 27 neurologically healthy individuals, (3) noted, that if the CiS or its secondary dimples closely approached the callosal sulcus, they usually separated area 24b dorsally from area 24a rostral to the genu. This sulcal variability can lead to systematic errors in the classification of these cytoarchitectonically defined sgACC areas. For example, if an intralimbic (secondary) sulcus is falsely classified as the cingulate sulcus delineating the ROI inferiorly, the defined area would only include subgenual BA 24a, but not BA 24b. To differentiate a secondary intralimbic sulcus (ILS) from the main branch of cingulate sulcus (CiS), several parasagittal slices had to be assessed. While the ILS usually appeared as a superficial dimple in mid-sagittal slices but disappeared within one or two slices from the midline, it was required for the CiS to be clearly evident in at least three parasagittal slices from the midline (53).

## 3 Results

### 3.1 Group comparisons

The study groups were well balanced in terms of their main potential confounding variables age (F_3, 80_ = 1.47 p = .27), gender (χ2_3 (n = 84)_ = 0.27, p = .97) and ICV (F_3, 80_ = .34, p = .80). Univariate ANOVAs assessing the difference between the four groups separately for the left and right sgACC volumes reached significance for left (F_3,80_ = 6.43, p <.001) and right hemisphere (F_(3,80)_ = 6.68, p <.001). To account for unexplained variance, group comparisons were then repeated under inclusion of ICV as covariate. The overall ANCOVA between groups reached significance for left (F_(3,79)_ = 6.15, p <.001) and right F_(3,79)_ = 6.63, p <.001 subgenual ACC volumes. *Post-hoc* analysis revealed smaller subgenual volumes in both the medicated (MDDm) and non-medicated (MDDu) group versus healthy controls (p = .001**;** p = .008) respectively for the left hemisphere. For the right hemisphere, the (MDDu) and BD group exhibited significantly lower subgenual volumes than healthy controls (p <.001; p = .004). These group comparisons remained significant after correcting with Bonferroni method with α level of significance set at p <.05. Excluding the outliers with unusually large sgACC volume in the left UPU group as well in the right BD group defined by the group-wise z-scores (|z| ≥ 2.58) from the full ANCOVA model did not change the overall results. **Table 3** shows the volumes of the entire dataset and detailed statistics on group comparisons.

**Table 3:** Absolute and corrected mean (± SD) sgACC volumes in mm3.

	MDDm(n=20)	MDDu (n=20)	BD (n=21)	HC (n=23)	Group comparison (n=84)	Correction for ICV (n=84)
Left sgACC	259.99 ± 60.96	283.75 ± 123.47	331.08 ± 135.63	401.89 ± 122.17	F_3,80_ = 6,43 ^A^ p <.001*	F_3,79 =_ 6,15^A^ p <.001*
Right sgACC	327.12 ± 136.75	269.16 ± 73.25	322.07 ± 118.85	400.98 ± 118.85	F_3,80_ = 6,68 ^A^ p <.001*	F_3,79 =_ 6,63 ^A^ p <.001*

*p ≤.05, ^A^AN(C)OVA statistics, MDDm, Major Depressive disorder (medicated); MDDu, Major Depressive disorder (unmedicated); BD, Bipolar disorder; HC, healthy controls; ICV, Intracranial volume. The p values listed in the tables are not corrected for multiple testing using Bonferroni correction.

### 3.2 Relationship between sgACC volume, clinical characteristics and medication

The sgACC volumes did not correlate with clinical characteristics such as duration of illness (years since 1st episode), the duration of the current episode (weeks since onset), number of depressive, non-depressive, or overall illness episodes in the patient groups except for the sgACC volumes in the right MDDm group, which correlated significantly with disease severity scores such as the BDI-II (r_20_ = 0.48, p = .004), HRSD (r_20_ = 0.35, p = .032) and IDS (r_20_ = 0.44, p = .007). However, after correction for multiple testing, no correlations remained significant.

The medicated patients were highly heterogeneous with respect to the psychopharmacological treatment within the 3 months prior to the neuroimaging. To test whether the sgACC volume was related to the different treatments, we grouped the medications according to their clinical use. Four homogeneous groups were identified as follows: antidepressant monotherapy (N = 9), antidepressants combined with sedative medication (i.e., benzodiazepines, anticonvulsants, or hypnotics; N = 7), antidepressants combined with either atypical neuroleuptics or lithium (N = 8) and therapies combining antidepressants with lithium plus atypical neuroleptics (N = 9). One-way ANOVAs yielded no effect for the type of medication on the sgACC volume (left sgACC: _F4, 30_ = 1.06, P = 0.4; right sgACC: F_4, 30_ = 1.27, P = 0.3).”.

## 4 Discussion

To our knowledge, this is the first morphometric MRI study using T1 maps obtained in high-resolution 7 Tesla MRI, providing submillimeter resolution for high precision volumetric procedures. While Drevets et al. (19) and studies replicating this protocol (54, 55) reported significant subgenual gray matter reductions in the sgACC mainly on the left hemisphere, other studies reported no differences in sgACC volumes between MDD patients and healthy controls (56–58). Meta-analyses conducted on existing volumetric studies with a ROI-based segmentation protocol indicated bilateral ACC volume reduction in patients with MDD compared to healthy controls, with less robust data on BD patients (9–11). These discrepant results reported in the literature are likely due to different definitions of structural landmarks with respect to how they define the sgACC region and, if present, the adjacent paracingulate gyrus, which is caused by relative expansion of the paracingulate sulcus (pcgs). ROI-based segmentation studies differed in terms of the inclusion or exclusion of this gyrus. Differences in patient samples, acquisition and analysis methods and a lack of uniform segmentation protocol may also contribute to these contradictory results. The extent of GMV reduction within our study groups is comparable to those reported in previous clinical protocols (19) ranging from 29-35% GMV reduction for left hemispheric MDD groups and 29-33% GMV reduction for the right hemispheric BD and MDDu groups relative to healthy controls measured in our ANCOVA model. Moreover, our reported GMV reductions are consistent with neuropathological findings reporting a loss of glial cells (4, 59), which is associated with a loss of neuropil hypothesized to arise secondarily from glutamate-induced excitotoxicity (60). Loss of neuropil as consequence of synaptic regression reported among mood disorders in ACC (61) concomitant with dendritic retraction is estimated to amount for the bulk of observed total GMV decrease, with glial cell reduction likely amounting only to a minor fraction of it. It is further hypothesized, that this synaptic deficit is one of the core pathologies responsible for poor function in the prefrontal-limbic network in MDD (62). Additionally, hypercortisolemia as a result of hyperactivity/disinhibition of the hypothalamic–pituitary–adrenal (HPA) axis has been frequently described as a hallmark of MDD (63, 64), leading to gradual decreases in tissue volume in MDD. Furthermore, medial prefrontal cortex and anterior cingulate contain high levels of glucocorticoid receptors and share extensive connections with structures within the hypothalamic-pituitary-adrenal (HPA) axis and have been implicated in regulation thereof (65, 66). Hyperactivity of the HPA axis has also been linked to the pathophysiology of BD (67), although data is less straightforward to interpret due to the neurotrophic effects of mood stabilizers such as lithium, which have been associated with normalized or increased GM volume in treatment responders (68, 69). While the volumetric data for sgACC reduction in BD is less robust, Hajek et al. (9) reported in their meta-analysis a trend for left subgenual volume decrease in bipolar patients with positive family history compared to healthy controls. While their sample size of included ROI studies (n=5) was low, we could only find a significant volume reduction in right hemispheric sgACC in BD patients.

The right hemispheric sgACC gray matter reduction in the MDDu group but not in the MDDm group is in line with findings of a meta-analysis by (11), in which patients without antidepressants had significantly smaller subgenual than patients taking antidepressants and healthy controls. Possible explanations for such an effect are increased neuroplasticity due to increased levels of neurotrophic factors, caused either by specific effects or secondary effects of antidepressants (70), or generally better illness course through earlier intervention seeking. Interestingly, this assumed neuroprotective effect was only pronounced in our patient samples for right hemispheric sgACC whereas for left hemispheric sgACC the medicated (MDDm) group exhibited even larger volume reductions than the MDDu group relative to healthy controls.

The strength of this study lies in hypothesis-driven ROI measurements in line with previously validated clinical protocols and careful controls against experimental bias through blinding the raters to the diagnoses. It includes a large sample size, and study groups were well balanced with respect to the main confounding variables age, sex, ICV, and disease severity. To avoid potential confounding variables, we included in the present study only individuals without neurological and cardiovascular comorbidities. There was no evidence that additional clinical or demographic factors contributed to sgACC volume differences in our patients.

Sulcal and gyral variability can be especially problematic for automated methods such as voxel-based morphometry (VBM), because they attempt to minimize differences in sulcal and gyral architecture by normalizing images to standard template space while also being prone to registration errors (71). Therefore, a ROI-based segmentation approach was chosen, while also benefitting from tissue probability maps (TPM) by means of predefining GM, WM and CSF tissue which reduced rater-dependent error and accounted for inter-individual variation of cortical folding patterns. Segmentation of sgACC GM tissue was performed manually to ensure consistency with known functional subdivisions defined in our segmentation protocol.

One of the limitations of our study is its cross-sectional design, in which patients with BD could be misdiagnosed with MDD based on their preliminary clinical course during their recruitment. Limitations of the computer-assisted segmentation algorithm must be considered. First, based on *in vivo* MRI data, our method cannot assess the microstructural (e.g. histological) validity of landmarks. Secondly, even in high-resolution 7 Tesla MR images, the sulcal boundaries of the cytoarchitectonically defined sACC areas cannot always be reliably matched to anatomic landmarks when accounting for inter-individual variability in gross brain morphology. Even though our defined sgACC ROI, apart from its anterior border, is encapsulated by sulcal structures filled with cerebrospinal fluid (CSF) tissue, these sulci could not always be adequately displayed in their full extent. Thus, their dorsal extension often had to be approximated, reducing the specificity with which our designated ROI may be interpreted as corresponding to a given Brodmann Area. This emphasizes the need for continued investigation into how sulcal variability is related to the configuration of functional fields within the cortex (72) and how probabilistic maps can further quantify variability in the position, size and extent of cytoarchitectonic areas (46). In conclusion, we have demonstrated left sgACC gray matter reduction in both the medicated and unmedicated MDD patient samples, and right sgACC gray matter reduction in the unmedicated depressed (MDDu) and bipolar (BD) patient samples, relative to healthy controls.

Gray matter volume reduction in the sgACC could serve as a potential biomarker in prediction and differential diagnosis of affective disorders, although further validation of these morphological differences is needed. With progress being made in functional connectivity MRI, the sgACC remains a promising target area for Intermittent theta-burst stimulation (iTBS) protocols (26), which have reported considerable remission outcomes in treatment-resistant depression by indirect stimulation of the sgACC.

As in many other studies, this investigation was cross-sectional and therefore provides limited information on the possible changes in sgACC volume over the course of illness. Ideally, future studies also incorporate a longitudinal design with follow-up measurements to be able to further distinguish cause from consequence.

## Data Availability

The datasets generated and/or analyzed during the current study are subject to privacy. Requests to access the datasets should be directed to the corresponding author.
