# Supplementary material for: Highfield imaging of the subgenual anterior cingulate cortex in uni- and bipolar depression
Source: Front Psychiatry. 2024 Oct 11;15:1462919. doi: 10.3389/fpsyt.2024.1462919 (PMC11502385; doi:10.3389/fpsyt.2024.1462919)
Supplement: Supplementary file 1 [file Table1.docx]

Protocol for semi-automatic segmentation of the subgenual Anterior Cingulate Cortex (sgACC)

**0. Segmentation conditions:**

- Closed sunblind and ambient light
- Standardized and fixed monitor position, contrast, angle and brightness
- Load T1map in Mipav(v11.08), open triplanar view
- Adjust the contrast range of the T1map greyscale image via LUT-Histogram menu to full intensity range {0-4095} with opacity level of Mask set at 50%
- Load mask onto T1map with both hemispheres being labeled by automatic tissue probability maps as follows:
- Label 1 - Grey matter (GM), Label 2 - White matter (WM), transparent voxels - Cerebrospinal Fluid (CSF)
- Block label 2 (WM) to prevent accidental segmentation of non-sgACC voxels and choose a label for segmentation

| **1. Compartmentalisation:** | |
| --- | --- |
|  |  |
|  | - Place ROI centrally, x15-x20 linear zoom is recommended for segmentation - Define anterior border in coronal view: Coming from rostrally, mark first slice of GM beneath the   rostral tip of the CC’s connected genu   - Define superior and inferior border in sagittal view: The ROI is bound superiorly by the CaS delineating   BA33 superiorly and inferiorly by the CiS (if CiS did not extend caudally beyond the genu, a line of its’  expected linear trajectory was traced).   - Differentiation between primary (Cingulate Sulcus) and secondary sulcal structures (Intralimbic sulcus) has to be   evaluated from their rostral course throughout at least three mid-sagittal slices to ensure correct classification   - Define posterior border in sagittal view:  1. Mark last slice of GM *prior* to the APS along its’ usual transition into CaS superiorly and into the CS inferiorly 2. If only the APS is apparent, the linear extension from its most superior end up to the CC’s Rostrum (as prev.   described by Spasojevic et al., 2011) delineated the ROI supero-posteriorly   1. For rare cases of APS not being clearly visible or being overshadowed by arterial artefacts, a vertical line from   the Rostrum down to the frontal lobe was drawn as a standardized posterior limit  **2. Coronal segmentation of sgACC gray matter:**   - Start segmentation at the first anterior slice in coronal plane by marking the boundary   voxels at the (1.) highest priority anatomical structure (see table 1) and connect those voxels  with maximum contrast to their neighbouring voxels sequentially to create a continuous line of voxel (outline)   - Include distinctively assignable grey matter voxels at bordering regions according to - their respective (3.) inclusion mode, decide conservatively in adjacent labeled WM/GM area,   liberally with respect to anatomical structure in adjacent labeled CSF area   - Continue to mark the GM for each slice until the APS delineates the ROI posteriorly from the adjacent   subcallosal gyrus (BA25)   - Check for any deviations from the underlying anatomical shape by fading mask in and out   **3. Sagittal and transversal segmentation of sgACC gray matter:**   - Subsequently mark the remaining GM area following the landmarks described in (table 2)   for sagittal plane and lastly (table 3) for the transversal plane:   - After marking each slice in sagittal plane, check for recursive deviations from the underlying   anatomical shape in coronal plane   - Consequently, check for deviations in coronal/sagittal planes after marking GM voxels in transversal plane |

**4. Review (triplanar)**

- Make sure segmentations trail anatomic structure by viewing unmarked sgACC in all planes.
- Reduce projections and indentions that deviate from the outline of the mask to the maximum length of one voxel. Erase arms above one voxel (or fill) single-voxel holes
- Check each correction and reject those that cause implausible changes in any plane
- All assignable GM voxels of the sgACC must be marked including occurrences of artifacts resulting from partial volume effects within the TPM where tissue evidently belongs to the GM-ROI

|  |  |
| --- | --- |

| **Coronal view** | **1.) Priority strucuture** | **2.) Automatic segmentation borders** | **3.) Inclusion mode** | **note** |
| --- | --- | --- | --- | --- |
| Rostral | first slice where a continuous CC is connecting both hemispheres | WM excluded | conservative decision (WM) |  |
| Superior | CaS (CSF)  CC (WM) | WM excluded | liberal decision (CSF),  conservative decision (WM) | one voxel tolerated as arm  at the outside border  Cave: exclude ACA |
| Inferior | CS or linear extension thereof (CSF) |  | liberal decision(CSF) | one voxel tolerated as arm  at the outside border |
| Lateral | CC (WM) | WM excluded | conservative decision (WM) | one voxel tolerated as arm  at the outside border |
| Medial | maximum median border: midline if anatomic structure not certainly visible |  | liberal decision (CSF) | one voxel tolerated as arm  at the outside border  Cave: exclude ACA |
| Caudal | last slice of voxel prior to the APS (CSF) (marked already as posterior border in sagittal view) |  | liberal decision (CSF) | Cave: exclude ACA |

| **Sagittal view** | **1.) Priority structure** | **2.) Automatic segmentation borders** | **3.) Inclusion mode** | **note** |
| --- | --- | --- | --- | --- |
| Rostral | first slice of voxel marked already as anterior border in coronal plane |  |  | |
| Superior | CaS (CSF),  CC (WM) | WM excluded | liberal decision (CSF),  conservative decision (WM) | one voxel tolerated as arm at the outside border  Cave: exclude ACA |
| Inferior | CS or linear extension thereof (CSF) |  | liberal decision with respect to anatomical structure (CSF) | one voxel tolerated as arm at the outside border |
| Lateral | CC (WM) | Wm excluded | conservative decision (WM) | one voxel tolerated as arm at the outside border |
| Medial | maximum median border: midline if anatomic structure not certainly visible |  | liberal decision (CSF) | one voxel tolerated as arm at the outside border  Cave: exclude ACA |
| Caudal | last slice of voxel prior to the APS (CSF) |  |  | |

| **Transversal view** | **1.) Priority structure** | **2.) Automatic segmentation borders** | **3.) Inclusion mode** | **note** |
| --- | --- | --- | --- | --- |
| Superior | voxel defined by marking in coronal view |  | conservative decision with respect to anatomical structure | one voxel tolerated as arm at the outside border |
| Inferior | voxel defined by marking in coronal view | WM excluded | conservative decision (WM) | one voxel tolerated as arm at the outside border |
| Rostral | first slice defined already as anterior border in coronal view |  | | |
| Lateral | CC (WM) | WM excluded | conservative decision (WM) | one voxel tolerated as arm at the outside border |
| Caudal | last slice defined already as posterior border in coronal view |  | | |
| Medial | maximum median border: midline if anatomic structure not certainly visible |  | liberal decision (CSF) | one voxel tolerated as arm at the outside border |
